# Supplementary material for: On the equivalence of one‐inflated zero‐truncated and zero‐truncated one‐inflated count data likelihoods
Source: Biom J. 2022 Aug 15;65(2):2100343. doi: 10.1002/bimj.202100343 (PMC10087693; doi:10.1002/bimj.202100343)
Supplement: Supplementary file 1 — Supporting Information. [file BIMJ-65-0-s001.zip › one_inflationR2.pdf]

Biometrical Journal **52** (2021) 61, zzz–zzz / DOI: 10.1002/bimj.200100000

On the equivalence of one-inflated zero-truncated and zero-truncated one-inflated count data likelihoods

Dankmar Böhning[*,1]

[1] Southampton Statistical Sciences Research Institute & Mathematical Sciences, University of Southampton, SO17 1BJ, UK

Received zzz, revised zzz, accepted zzz

One-inflation in zero-truncated count data has recently found considerable attention. There are currently two views in the literature. In the first approach, the untruncated model is considered as one-inflated whereas in the second approach the truncated model is viewed as one-inflated. Here, we show that both models have identical model spaces as well as identical maximum likelihoods. Consequences for population size estimation are illuminated and and the findings are illustrated at hand of two case studies.
Keywords: capture-recapture; one-inflation; inflation of singletons; zero-truncation; population size estimation.

Key words: capture-recapture; Inflation of singletons; One-inflation; Population size estimation; Zero-truncation.

1 Introduction

In a nutshell, this paper makes the following point. The use of the one-inflated zero-truncated count model approach has become quite common as it is easy to deal with due to the fact that one inflation can be coped with by simply truncating the ones, in addition to the zeros (Böhning and Ogden, 2021). The zero-truncated one-inflated model has been viewed thus far as a different model because, for example, both models have different maximum likelihood estimators. The latter is also more difficult to treat as the weight for the one-inflation component enters non-linearly. The result in this work shows that both models are equivalent from an inference point of view and there is no loss in working with the simpler, one-inflated zero-truncated model.

To be more precise, we consider a count random variable Y taking values in the set of non-negative integers. The application we have in mind is that Y represents the number of identifications in a capture-recapture experiment or study. We assume a generic count density $p(y;\theta)$ for Y where y takes values in the set of non-negative integers and θ is real-valued parameter or vector. We call this the *base-distribution*. Typical examples of the base-distribution include the Poisson, geometric, binomial or negative-binomial distribution among others. In applications we sometimes see the occurrence of a large amount of ones, the so-called singletons, relative to the base distribution. In this case we speak of the occurrence of *one-inflation*. One-inflation models have recently found a lot of attention (see Böhning and Ogden 2021, Böhning and van der Heijden 2019, Böhning and Friedl 2021, Godwin 2017, Godwin and Böhning 2017, Godwin 2019). Such a phenomenon of one-inflation can be easily addressed using the model

$$p_1(y;\theta,w) = (1-w)I_1(y) + w\, p(y;\theta), \tag{1}$$

where y takes values in the set of non-negative integers and $I_1(.)$ is the indicator function defined as $I_1(y) = 1$ if $y = 1$ and 0 otherwise, and $w \in (0,1]$ is a weight. $(1-w)$ represents the amount of one-inflation (the *extra ones*) in the population, relative to the base-distribution $p(y;\theta)$.

*Corresponding author: e-mail: d.a.bohning@soton.ac.uk, Phone: +0044-23-8059-6712

© 2021 WILEY-VCH Verlag GmbH & Co. KGaA, Weinheim

In uni-list capture-recapture settings the number Y of identifications of units of the target population with the characteristic of interest is positive as those with zero identifications do not occur in the observed sample. Hence, we need to consider the associated zero-truncated distribution. This brings the topic into the area of capture-recapture methods (see for example Böhning *et al.* (2018); McCrea and Morgan (2014)). For a general discrete mass probability function $g(y)$ for $y = 0, 1, 2, ...$ the associated zero-truncated probability mass function is given as $g(y)/(1 - g(0))$ for $y = 1, 2, ...$ Hence the zero-truncated discrete probability mass function associated with (1) is

$$p_{1+}(y;\theta,w) = \frac{(1-w)}{1-w\,p(0;\theta)} I_1(y) + \frac{w}{1-w\,p(0;\theta)} p(y;\theta), \tag{2}$$

for $y = 1, 2, ...$ As we zero-truncate a one-inflation model we call (2) the *zero-truncated one-inflated* model associated with the base distribution $p(y; \theta)$.

In a different approach the one-inflation is only applied to the positive part of the distribution such as

$$\tilde{p}_1(y;\theta,w) = \begin{cases} p(0;\theta) & \text{if } y = 0 \\ (1-w)(1-p(0;\theta))I_1(y) + w\,p(y;\theta) & \text{if } y > 0 \end{cases} \tag{3}$$

where $y = 0, 1, 2, ...$ Now, the zero-truncated discrete probability mass function associated with (3) is provided as

$$p_{+1}(y;\theta,w) = (1-w)I_1(y) + \frac{w}{1-\ p(0;\theta)} p(y;\theta), \tag{4}$$

which we can think of as the one-inflated distribution associated with the zero-truncated distribution $\frac{p(y;\theta)}{1-\ p(0;\theta)}$. Hence we call (4) the *one-inflated zero-truncated* model associated with the base distribution $p(y; \theta)$. The wording is conventional, so in a way arbitrary. However, the logic here is as follows. Model (1) assumes that the base distribution experiences one-inflation which becomes truncated in the observational model (2). This is the zero-truncated one-inflated model (ZTOI). In contrast, model (4) assumes that the observational, zero-truncated model is one-inflated. This is the one-inflated zero-truncated model (OIZT).

Any inference must refer to the observable discrete mass probability functions (2) and (4). As (2) and (4) are different models, interest arises how they are connected.

Case-study on dice snake in Graz (Austria). Before we come to the main objectives of the paper let us illustrate the issue with data from a case study presented in Böhning and Friedl (2021) on estimating the population size of dice snakes in a closed area at the river Mur in Graz (Austria). The work was motivated by a resettlement project of the population due to the development of a water power plant in the vicinity of the living ground of the dire snakes. The major question here was: how many dice snakes are there? We focus here on the year 2014 in which there were 31 capture occasions during the year. As above, Y denotes the identification count per dice snake. The empirical distribution of Y is provided in Table 1. The frequency f_0 of dice snakes never sighted is unknown and aimed to be estimated. The concern here is that the frequency $f_1 = 59$ of singletons is large in size, potentially too large to be compatible with a conventional count distributional model. This could lead to overestimation bias in the population size estimator of dice snake prevalence in the target area (Böhning and Friedl 2021).

Table 1 Frequency of count of sightings per dice snake in the target area in 2014

count y of sightings	0	1	2	3	4	5	n
frequency f_y	-	59	8	1	1	1	70

This paper has the following objectives:

© 2021 WILEY-VCH Verlag GmbH & Co. KGaA, Weinheim

1) we will show that (2) and (4) have identical maximum likelihoods,
2) and also show that there exists a one-to-one mapping that transfers respective maximum likelihood estimates into each other,
3) and also demonstrate how population size estimates based on the likelihoods (2) and (4), respectively, can be achieved by means of Horvitz-Thompson estimation.

The following section 2 contains the central results.

2 Main results

We will consider the following two sets. The first set is related to (4) and defined as

$$\Gamma = \{(p_{+1}(1;\theta,w),\cdots,p_{+1}(y;\theta,w),\cdots)'|w \in (0,1], \theta \in \Theta\}. \tag{5}$$

Here $'$ refers to transposing a vector and Θ is the parameter set, a subset of the real line or finite-dimensional real space. The second set is related to (2) and defined as

$$\tilde{\Gamma} = \{(p_{1+}(1;\theta,w),\cdots,p_{1+}(y;\theta,w),\cdots)'|w \in (0,1], \theta \in \Theta\}. \tag{6}$$

We have the following

Theorem 2.1

$$\tilde{\Gamma} = \Gamma$$

Proof. We show that if

$$p_{+1}(1;\theta,w),\cdots,p_{+1}(y;\theta,w),\cdots)' \in \Gamma$$

then also

$$p_{+1}(1;\theta,w),\cdots,p_{+1}(y;\theta,w),\cdots)' \in \tilde{\Gamma}.$$

Now, for any positive integer y,

$$p_{+1}(y;\theta,w) = (1-w)I_1(y) + \frac{w}{1-\ p(0;\theta)}p(y;\theta)$$

can be written as

$$p_{1+}(y;\theta,\tilde{w}) = \frac{(1-\tilde{w})}{1-\tilde{w}\,p(0;\theta)}I_1(y) + \frac{\tilde{w}}{1-\tilde{w}\,p(0;\theta)}p(y;\theta)$$

where

$$\tilde{w} = \frac{w}{1-p(0;\theta)+w\,p(0;\theta)} \in (0,1).$$

This shows that $p_{+1}(1;\theta,\tilde{w}),\cdots,p_{+1}(y;\theta,\tilde{w}),\cdots)' \in \tilde{\Gamma}$.

Now, we show that if

$$p_{1+}(1;\theta,\tilde{w}),\cdots,p_{1+}(y;\theta,\tilde{w}),\cdots)' \in \tilde{\Gamma}$$

then also

$$p_{1+}(1;\theta,\tilde{w}),\cdots,p_{1+}(y;\theta,\tilde{w}),\cdots)' \in \Gamma.$$

Again, for any positive integer y,

$$p_{1+}(y;\theta,\tilde{w}) = \frac{(1-\tilde{w})}{1-\tilde{w}\,p(0;\theta)}I_1(y) + \frac{\tilde{w}}{1-\tilde{w}\,p(0;\theta)}p(y;\theta)$$

© 2021 WILEY-VCH Verlag GmbH & Co. KGaA, Weinheim

can be written as

$$p_{+1}(y;\theta,w) = (1-w)I_1(y) + \frac{w}{1-\ p(0;\theta)}p(y;\theta),$$

where

$$w = \frac{\tilde{w}(1-p(0;\theta))}{1-\tilde{w}\,p(0;\theta)} \in (0,1).$$

This shows that $p_{1+}(1;\theta,\tilde{w}),\cdots,p_{1+}(y;\theta,\tilde{w}),\cdots)' \in \Gamma$ and ends the proof. □

In Figure 1 we see an illustration of the result above for singletons (those units where $y = 1$) and doubletons (those units where $y = 2$).

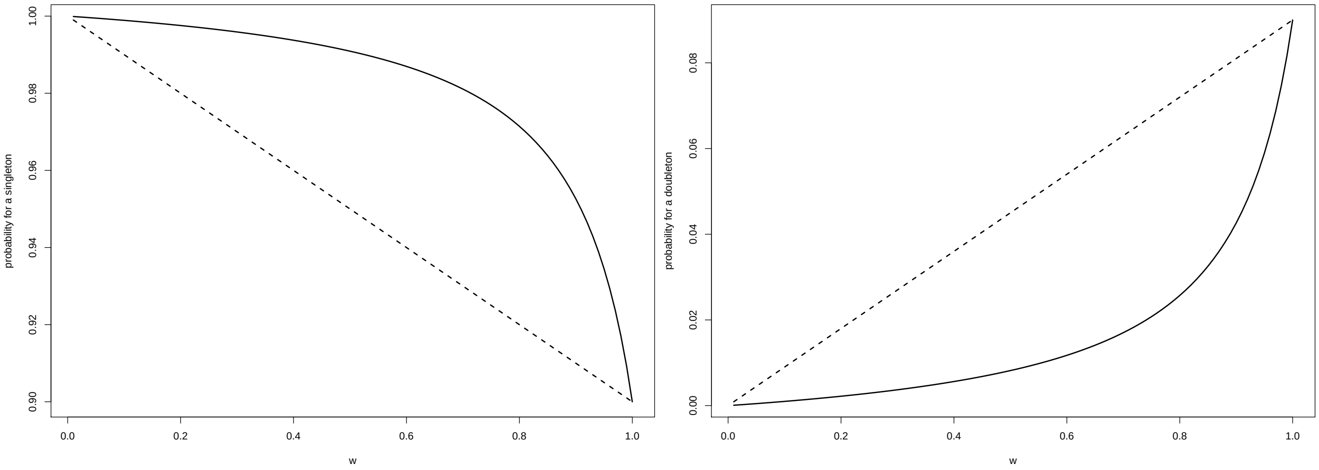

Figure 1 Probability for a singleton (left panel) and a doubleton (right panel) according to Γ (dashed) and according to $\tilde{\Gamma}$ (solid) for fixed $\theta = 0.9$ and w varying in $(0,1]$

Suppose that a random sample $y_1,\cdots,y_n$ of size n is available. Also, let f_y denote the frequency of those y_i in the sample $y_1,\cdots,y_n$ such that $y_i = y$. So, f_1 is the frequency of singletons, f_2 the frequency of doubletons, f_3 the frequency of tripletons, and so forth. Then the log-likelihood with respect to (2) can be written as

$$\tilde{\ell}(\theta,w) = \sum_{y=1}^{m} f_y \log[p_{1+}(y;\theta,w)], \tag{7}$$

where m is the largest count observed and $(\theta,w)' \in \tilde{\Gamma}$. The log-likelihood with respect to (4) is given as

$$\ell(\theta,w) = \sum_{y=1}^{m} f_y \log[p_{+1}(y;\theta,w)], \tag{8}$$

where $(\theta,w)' \in \Gamma$. As Γ and $\tilde{\Gamma}$ coincide we have the following

Theorem 2.2

$$sup_{(\theta,w)'\in\Theta\times(0,1]}\ \tilde{\ell}(\theta,w) = sup_{(\theta,w)'\in\Theta\times(0,1]}\ \ell(\theta,w)$$

© 2021 WILEY-VCH Verlag GmbH & Co. KGaA, Weinheim

Theorem 2.2 means that maximum likelihoods agree irrespective which model, (2) or (4), they come from. This also means that there is no way to identify, on the basis of the likelihoods, from which of the two models the sample has arisen from. An illustration of the result is provided in Figure 2 using the drink-driving data of Britain.

It also follows from the invariance principle for maximum likelihood estimation that maximum likelihood estimates can be easily found for one model if they are available for the other. So, if $\hat{w}$ and $\hat{\theta}$ are maximum likelihood estimates for (8) then $\hat{\theta}$ and

$$\frac{\hat{w}}{1 - p(0;\hat{\theta}) + \hat{w}\,p(0;\hat{\theta})}$$

are the associated maximum likelihood estimates for (7). Vice versa, if $\hat{w}$ and $\hat{\theta}$ are maximum likelihood estimates for (7) then $\hat{\theta}$ and

$$\frac{\hat{w}(1 - p(0;\hat{\theta}))}{1 - \hat{w}\,p(0;\hat{\theta})}$$

are the associated maximum likelihood estimate for (8).

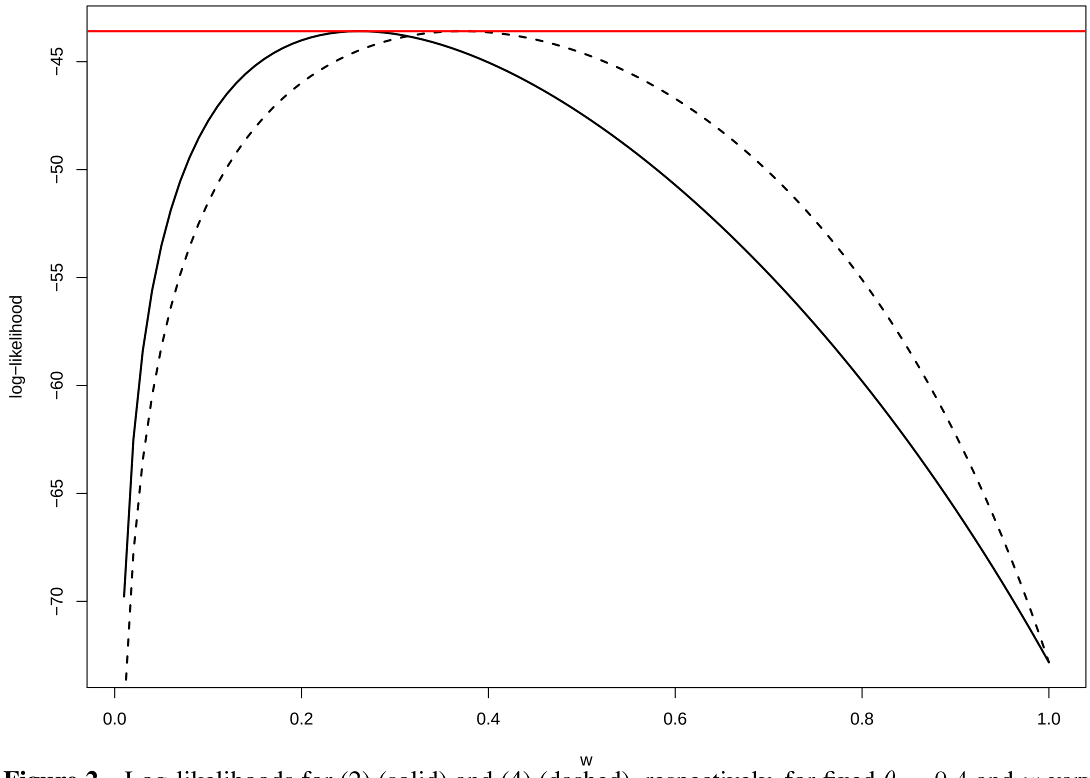

Figure 2 Log-likelihoods for (2) (solid) and (4) (dashed), respectively, for fixed $\theta = 0.4$ and w varying in $(0, 1]$ using the dice snake data of Graz (Austria) in 2014

A benefit of Theorems 2.1 and 2.2 is that it does not matter which one of the models, (2) or (4), is used as they are equivalent. However, model (4) is mathematically easier to deal with as the weight occurs linearly, and also model (4) fits better to the existing theory of one-inflation models of zero-truncated distributions

© 2021 WILEY-VCH Verlag GmbH & Co. KGaA, Weinheim

Böhning and Friedl (2021). Although the results of this section are of interest in themselves the question arises how they affect population size estimation which is addressed in the next section 3.

3 Population size estimation

We have said that for a general discrete mass probability function $g(y)$ for $y = 0, 1, 2, ...$ the associated zero-truncated probability mass function is given as $g(y)/(1 - g(0))$ for $y = 1, 2, ...$ So, given a sample of size n the Horvitz-Thompson estimator of $E(f_0)$ is given by

$$\hat{f}_0 = n \frac{g(0)}{1 - g(0)}. \tag{9}$$

The estimator (9) is unbiased as

$$E(\hat{f}_0) = \frac{g(0)}{1 - g(0)} E(n) = \frac{g(0)}{1 - g(0)} N(1 - g(0)) = Ng(0) = E(f_0),$$

assuming that n is binomial with size parameter N, the true population size, and event probability $1 - g(0)$.

In model (3) we have that $g(0)$ is simply $p(0; \theta)$ as the inflation parameter w does not occur here. Hence

$$\hat{f}_{0,\text{OIZT}} = n \frac{p(0; \theta)}{1 - p(0; \theta)}. \tag{10}$$

In model (1) we have that $g(0) = wp(0; \theta)$ so that the probability for count zero involves the inflation parameter w. If we replace w by its maximum likelihood estimate $\frac{1-f-1/n}{1-p(1;0)-p(0;\theta)f_1/n}$ we find

$$\hat{f}_{0,\text{ZTOI}} = (n - f_1) \frac{p(0; \theta)}{1 - p(0; \theta) - p(1; \theta)}. \tag{11}$$

The result can be interpreted in the following way. Consider the reduced sample size $n_1 = n - f_1$, the original sample size reduced by the number of singletons, then n_1 is binomial with size parameter N and event probability $1 - p(0; \theta) - p(1; \theta)$. We formally state this as the following

Theorem 3.1

$$\hat{f}_{0,ZTOI} = n_1 \frac{p(0; \theta)}{1 - p(0; \theta) - p(1; \theta)}$$

P r o o f. We abbreviate $p_0 = p(0; \theta)$ and $p_1 = p(1; \theta)$ with θ known. Now, we use that the maximum likelihood estimate of $w$is $\tilde{w} = \frac{1-f_1/n}{1-p_1-p_0 f_1/n}$ so that

$$\hat{f}_0 = n \frac{\frac{1-f_1/n}{1-p_1-p_0 f_1/n} p_0}{1 - \frac{1-f_1/n}{1-p_1-p_0 f_1/n} p_0}$$

$$= n \frac{(1 - f_1/n) p_0)}{1 - p_1 - p_0)} = (n - f_1) \frac{p_0}{1 - p_1 - p_0}.$$

□

The associated Horvitz-Thompson estimators of the population size N are then given by

$$\hat{N}_{0,\text{OIZT}} = \underbrace{n_1 + f_1}_{n} + \hat{f}_{0,\text{OIZT}}$$

and

$$\hat{N}_{0,\text{ZTOI}} = n + \hat{f}_{0,\text{ZTOI}}.$$

Note that the population size estimator does not involve w and solely depends on θ. In practice, a plug-in estimator for θ needs to be used.

© 2021 WILEY-VCH Verlag GmbH & Co. KGaA, Weinheim

4 Applications

Case-study on dice snake in Graz (Austria) (continued). The major aim of the paper is to show the equivalence of the zero-truncated one-inflated with the one-inflated zero-truncated distributional model. However, it is also important to see how this result can be used. We use the latter model for the data of the case study on estimating the population size of dice snakes in a closed area at the river Mur in Graz (Austria). We choose the geometric distribution $p(y;\theta) = \theta(1-\theta)^y$ $(y = 0, 1, 2, ...)$ as it turns out to be well fitting in this situation (Böhning and Friedl 2021). In addition, it can be shown that maximum likelihood estimation in one-inflated zero-truncated models can be accomplished by considering maximum likelihood estimation in zero-one truncated models (Böhning and Ogden 2021). The zero-one truncated geometric distribution is given by $\theta(1-\theta)^{y-2}$ for $y = 2, 3, ...$ and its maximum likelihood estimate by $\hat{\theta}_1 = \frac{n-f_1}{S-n} = (70-59)/(87-70) = 0.6471$ where $S = \sum_x xf_x$. It is shown in Böhning and Ogden (2021) that the maximum likelihood for the one-inflated zero-truncated model (see also (8)) is given by

$$\log L_1 = (S+f_1-2n)\log(1-\hat{\theta}_1)+(n-f_1)\log(\hat{\theta}_1)+f_1\log(f_1/n)+(n-f_1)\log(1-f_1/n) = -41.48.$$

Here the merit of the one-inflated zero-truncated model emerges as the likelihood can be achieved by simply truncating counts of zero (naturally occurring) as well as truncating ones. Given $\hat{\theta}_1 = \frac{n-f_1}{S-n} = 0.6471$, the maximum likelihood estimate of w is provided as $\hat{w} = \frac{1-f_1/n}{1-\hat{\theta}} = 0.4452$ (Böhning and Ogden 2021).

For illustration, we also provide the zero-truncated one-inflated maximum likelihood (see also (7)) as

$$\log \tilde{L}_1 = (S-f_1)\log(1-\hat{\theta})+(n-f_1)\log\hat{\theta}+f_1\log\left(\frac{1-\hat{\tilde{w}}+\hat{\tilde{w}}\hat{\theta}(1-\hat{\theta})}{1-\hat{\tilde{w}}\hat{\theta}}\right)+(n-f_1)\log\left(\frac{\hat{\tilde{w}}}{1-\hat{\tilde{w}}\hat{\theta}}\right)$$

which also takes on the value -41.48. Note that $\hat{\theta}$ agree for both models, only the weight estimates differ with $\hat{\tilde{w}} = \hat{w}/(1-\hat{\theta}+\hat{w}\hat{\theta})$ where $\hat{w}$ is the maximum likelihood estimate from the one-inflated zero-truncated model. These results are also summarized in Table 2.

We have also provided confidence intervals for population size estimates in Table 2. These are based on a non-parametric bootstrap as follows. Based on the estimated $\hat{N}$, random samples of size $\hat{N}$ (rounded to the next integer) are drawn from the distribution of interest using the sampling probabilities $\hat{f}_0/\hat{N}$, $f_1/\hat{N}$, $f_2/\hat{N}$, ..., and for each sample the population size estimate determined. As the distribution of $\hat{N}$ is highly skewed we use the log-transformation first to calculate approximate normal-based 95% confidence intervals and then use the anti-log to achieve lower and upper confidence limits, respectively, for a 95% confidence interval for N. The details are again provided in Table 2.

Table 2 Log-likelihood, AIC, maximum likelihood estimates of θ and w (or $\tilde{w}$) for the one-inflated zero-truncated model (OIZT) given in (4), the zero-truncated one-inflated (ZTOI) given in (2) and the non-inflated model in the case of the dice snake data of Graz followed by population size estimates with 95% confidence intervals based on a non-parametric bootstrap

model	log-likelihood	AIC	$\hat{\theta}$	weight	$\hat{N}$	95% CI
OIZT	-41.48	86.96	0.6471	0.4452 ($\hat{w}$)	198	123 – 342
ZTOI	-41.48	86.96	0.6471	0.6946 ($\hat{\tilde{w}}$)	127	74 – 248
no inflation	-42.97	87.95	0.8046	-	358	247 – 547

Ignoring one-inflation in the zero-truncated geometric model entirely, we find $\hat{\theta}_0 = n/S$ and the associated maximum likelihood

$$\log L_0 = (S-n)\log(1-\hat{\theta}_0) + n\log(\hat{\theta}_0) = -42.97.$$

© 2021 WILEY-VCH Verlag GmbH & Co. KGaA, Weinheim

The likelihood ratio tests delivers 2.90 with p-value of 0.0838. The corresponding values of the Akaike information criterion are 86.96 (for the one-inflated zero-truncated model) and 87.95 for the zero-truncated model without one-inflation, indicating borderline evidence for one-inflation. It is also interesting to see the effect of ignoring one-inflation in the population size estimates. For the model with one-inflation we find $\hat{N} = 198$ under OIZT sampling and $\hat{N} = 127$ under ZTOI sampling, whereas for the model ignoring one-inflation we find $\hat{N} = 358$ which is considerably higher than the estimates without one-inflation. The associated 95% confidence intervals are given in Table 2. As the dice snake data set has small sample size it is not surprising that the confidence show considerable overlap. Nevertheless, the approach allowing for one-inflation seems to provide the more realistic estimate of the size of the dice snake population as the non-inflated model is prone to overestimation of the size. This underlines the importance of considering one-inflation, especially when considering population size estimation.

Case-study on family violence in the Netherlands in 2009. The previous case-study is characterized by a weak evidence for one-inflation which nevertheless had considerable impact on the population size estimate. In this case study, there is much stronger evidence for one-inflation. Family violence data for the Netherlands in the year 2009 provided by van der Heijden et al. (2014)(also discussed in Böhning et al. 2019). Here the perpetrator study is reported with the data given in Table 3. There were 15,169 perpetrators identified being involved in a domestic violence incident exactly once, 1957 exactly twice, and so forth. In total, there were 17,662 different perpetrators identified in the Netherlands for 2009. The data represent the Netherlands except the police region for The Hague. The results of the analysis for these data is presented in Table 4. Again, the geometric was used (as suggested in Böhning et al. 2019) as base distribution. Here there is clear evidence for one-inflation and the confidence intervals for the population size estimates are narrow and do not overlap. In a nutshell, the result implies that there are about 3.5 to 4.5 times more perpetrators in total than have been observed (based on the respective one-inflation model). Ignoring one-inflation, the factor would about 7.5 which is likely an overestimate.

Table 3 Frequency of the number of times perpetrators have been identified in a domestic violence incident in the Netherlands in the year 2009

count y of identifications	0	1	2	3	4	5	6	n
frequency f_y	-	15,169	1957	393	99	28	16	17,662

Table 4 Log-likelihood, AIC, maximum likelihood estimates of θ and w (or $\tilde{w}$) for the one-inflated zero-truncated model (OIZT) given in (4), the zero-truncated one-inflated (ZTOI) given in (2) and the non-inflated model in the case of the family violence data followed by population size estimates with 95% confidence intervals based on a non-parametric bootstrap

model	log-likelihood	AIC	$\hat{\theta}$	weight	$\hat{N}$	90% CI
OIZT	-8926.91	17857.82	0.7713	0.6173 ($\hat{w}$)	77,244	73,602 – 80,996
ZTOI	-8926.91	17857.82	0.7713	0.8759 ($\hat{\tilde{w}}$)	54,443	50,741 – 58,683
no inflation	-9000.12	18002.23	0.8453	-	114,180	111,288 – 117,093

Further examples can be found in the supplementary material.

5 Discussion

We end the paper with a short discussion. Model (1) can be thought of a mixture model with two subpopulations. In one subpopulation the baseline distribution $p(y;\theta)$ holds, in the other a one-point distribution is giving all its mass to the singletons. In the latter subpopulation there is no positive probability given to any

© 2021 WILEY-VCH Verlag GmbH & Co. KGaA, Weinheim

count other than the singletons. Model (4) can be viewed as model one-inflation model given the knowledge of a positive capture count Y. We have seen that both models lead to the same inference with repsect to the likelihoods involved. When it comes to population size estimation by means of Horvitz-Thompson it matters which of the two sampling models holds.

Hence it is important to view how one-inflation occurs. For example, let us look at a misclassification perspective. It can be thought that some of the singletons are in fact doubletons, triplets, ... but have been counted as singletons. For example, it could be speculated that some of the drivers identified once have been drink-driving more than once but have not been identified at these occasions. In an extreme situation, this means that the over-inflated units are redistributed to the first distribution $p(y;\theta)$. This would not change inference w.r.t. θ but w.r.t. the population size. In this case, it would be more meaningful to use $\hat{N} = n/[1-p(0,\theta)]$ as all the inflated counts would in some way belong to the base distribution and would need to be up-weighted with the Horvitz-Thompson estimator. Hence here OIZT-sampling would be appropriate,

Yet, in another scenario that can be envisioned, doubletons, for example, are not identified as being the same unit counted twice but as different units and, hence, lead to an inflation of ones. This scenario of undermatching will unlikely be true for the drink-driving study, but if we imagine a biodiversity study where it might be only possible to identify identical genetic material to a certain percentage degree, this case could occur. Bunge *et al.* (2012) consider such a situation:

> This is the case, for example, when dealing with high-throughput DNA sequencing data, which are prone to errors of various types. These errors may arise at various stages, in particular the identification of the sequences and the clustering algorithms used to combine sequences into clusters or taxa may be questionable. The end result is that the number of low frequency counts especially the singletons (f1) may be artificially inflated compared to what would be obtained by a data-collection process with a lower error rate.

In this case it is unclear what f_1 really represents. How many are true singletons, how many are doubletons, tripletons ....? Hence a population size estimator would be difficult to determine. However, assuming that the frequency of doubletons, tripletons, ..., in other words the frequencies $f_2, f_3, \ldots$ are correct (but not f_1), a valid estimator of the hidden or unobserved units can be given using $\hat{f}_{0,\text{OIZT}}$ as it only builds on $n_1 = n - f_1$. This could still provide useful information. In closing, it is important to develop an understanding of the mechanisms which lead to the occurrence of one-inflation.

References

[1] BÖHNING, D. & FRIEDL, H. (2021). Population size estimation based upon zero-truncated, one-inflated and sparse count data. *Statistical Methods & Applications*, DOI: 10.1007/s10260-021-00556-8.

[2] BÖHNING, D. & VAN DER HEIJDEN, P. G. M. (2019). The identity of the zero-truncated, one-inflated likelihood and the zero-one-truncated likelihood for general count densities with an application to drink-driving in Britain. *Annals of Applied Statistics* **13**, 1198-1211.

[3] BÖHNING, D., VAN DER HEIJDEN, P. G. M., & BUNGE, J. (2018). *Capture-Recapture Methods for the Social and Medical Sciences.* Boca Raton, FL: Chapman & Hall/CRC.

[4] BÖHNING, D., KASKASAMKUL, P. , & VAN DER HEIJDEN, P. (2019). A modification of Chao's lower bound estimator in the case of one-inflation. *Metrika* **82**, 361–384.

[5] BÖHNING, D. & OGDEN, H. E. (2021). General flation models for count data. *Metrika* **84**, 245-261.

[6] BUNGE, J., BÖHNING, D., ALLEN, H., & FOSTER, J.A. (2012). Estimating population diversity with unreliable low frequency counts. *Biocomputing 2012*, 203–212, doi.org/10.1142/9789814366496_0020.

[7] GODWIN, R. T. (2017). One-inflation and unobserved heterogeneity in population size estimation. *Biometrical Journal* **59**, 79-93.

[8] GODWIN, R. T. (2019). The one-inflated positive Poisson mixture model for use in population size estimation. *Biometrical Journal* **61**, 1541-1556.

© 2021 WILEY-VCH Verlag GmbH & Co. KGaA, Weinheim

[9] GODWIN, R. T. & BÖHNING, D. (2017). Estimation of the population size by using the one-inflated positive Poisson model. *Journal of the Royal Statistical Society: Series C (Applied Statistics)* **66**, 425-448.

[10] MCCREA, R. S. & MORGAN, B. J. (2014). *Analysis of Capture-Recapture Data.* Boca Raton, FL: Chapman & Hall/CRC.

[11] VAN DER HEIJDEN, P. G. M., CRUYFF, M. AND BÖHNING, D. (2014). Capture-recapture to estimate crime populations. In: G.J.N. Bruinsma and D.L. Weisburd (eds.). *Encyclepedia of Criminology and Criminal Justice.* Berlin: Springer, 267 - 278.

Acknowledgements I am grateful to Herwig Friedl (Technical University of Graz) for the many interesting discussions on the topic.

Conflict of Interest

The author has declared no conflict of interest.

© 2021 WILEY-VCH Verlag GmbH & Co. KGaA, Weinheim
